# Introduction of the Personal Domain in Water Sanitation and Hygiene (WASH), a New Approach to Identify Missing Health Impacts

**DOI:** 10.3390/tropicalmed8050252

**Published:** 2023-04-26

**Authors:** Peter Kjær Mackie Jensen, Zenat Zebin Hossain, Rebeca Sultana, Jannatul Ferdous, Sara Almeida, Anowara Begum

**Affiliations:** 1 Copenhagen Center for Disaster Research, Global Health Section, Department of Public Health, University of Copenhagen, Øster Farimagsgade 5, Building 22, 1014 Copenhagen, Denmark; 2 Department of Microbiology, University of Dhaka, Dhaka 1000, Bangladesh; 3 Department of Public Health, School of Pharmacy and Public Health, Independent University, Dhaka 1229, Bangladesh; 4 icddr,b, Dhaka 1212, Bangladesh; 5 Institute of Health Economics, University of Dhaka, Dhaka 1000, Bangladesh

**Keywords:** personal domain, domestic domain, public domain, pathogen transmission, drinking water, WASH, health impact, F-diagram, transmission routes

## Abstract

The water sanitation and hygiene (WASH) sector has provided beneficiaries in low and middle-income countries with latrines and clean water for decades. However, we still need good evidence documenting the expected health impact. This paper investigates why we lack this evidence and ways to move forward. Using mTEC agar, we monitored *E. coli* contamination on selected “hotspot” surfaces within the kitchen environments of 32 low-income households in Dhaka, Bangladesh, every six weeks for two years. Despite being washed, the highest average contamination was found on food plates, at 253 cfu/10 cm^2^, followed by cutting knives, with 240 cfu/10 cm^2^. The drinking vessel surfaces and the latrine doorknobs had the lowest contaminations, with *E. coli* means of 167 and 73 cfu/10 cm^2^, respectively. These findings imply a need to measure an individual’s pathogen exposure as close to the mouth as possible to estimate the true pathogen exposure. The paper proposes introducing the new “personal domain”—the point of consumption—as the physical sphere in which WASH interventions should be assessed. With this approach, we can observe and quantify the different pathogen exposure routes and, with this, further improve WASH interventions.

## 1. Introduction

The global death toll from diarrheal diseases has declined in recent decades. Still, it remains exceptionally high, with an estimated 1.57 million deaths annually, of which one-third corresponds to those under the age of 5 years [1]. Underneath these staggering numbers is an unknown number of cases of severe and non-severe diarrhea, more than a billion, which significantly impact the societal and the individual household economies, particularly in lower-middle income countries such as Bangladesh [2,3].

Diarrheal disease is primarily linked to the ingestion of fecal pathogens transmitted by the routes clearly illustrated in the F-diagram proposed in 1958 by Wagner and Lanoix [4]. This simple diagram still serves as a road map to identify where to interrupt the different fecal–oral transmission routes to prevent the spread of diarrheal diseases. Until the late 2010s, we believed ourselves to have a good understanding of these enteric pathogens’ transmission routes and how to break them with relatively “simple” water sanitation and hygiene (WASH) interventions, such as clean drinking water, hygiene education, handwashing, and provision of latrines [5,6,7]. Without challenging the scientific background, this presupposition has set the direction for prioritizing interventions and strategies within the WASH sector, in both developmental and humanitarian contexts. One of the reasons for this is probably that the WASH sector is deeply rooted in solid historical evidence of the perceived consequence of Dr. John Snow’s removal of the pump handle in 1854, which forever linked cholera and, therefore, diarrhea to drinking water quality [8]. Keeping the historical legacy in mind, it is noteworthy that except for the positive health impact of handwashing, improving water quality and sanitation has rarely been documented in the literature for low-resource settings [9]. The general notion was that the lack of large, extensive, well-designed multi-arm studies of longer duration was the reason for the missing documentation of the effect and the final proof of the water quality–health causality. However, in 2019, three such comprehensive and highly rigorous randomized controlled trials of water quality (drinking water chlorination), sanitation (upgraded sanitation), and hygiene (promotion of handwashing with soap) in low-income households (parallel studies on WASH benefits in Bangladesh and Kenya and the similar Sanitation Hygiene Infant Nutrition Efficacy (SHINE) study in Zimbabwe) reported back [10,11,12]. Despite the expectations, none of the studies showed any effects on linear growth in children; moreover, only the Bangladesh study showed a modest impact on pediatric diarrhea. It remains unclear why the studies did not obtain the expected reduction in diarrhea [13], but the authors [14] discussed the possible problem of not including domestic water availability in the studies. The WASH benefits households in Bangladesh had wells and, therefore, easy access to domestic and drinking water, while in Kenya, they had to transport water to the house manually. This could potentially be an important factor, as the lack of an adequate quantity of domestic water has been linked to a household’s ability to maintain an acceptable hygiene level [15,16,17]. However, studies rarely report on domestic water use in rural and low-income communities, as substantial methodological challenges exist in monitoring and quantifying the often-non-metered water use [18,19].

**Figure 1:**
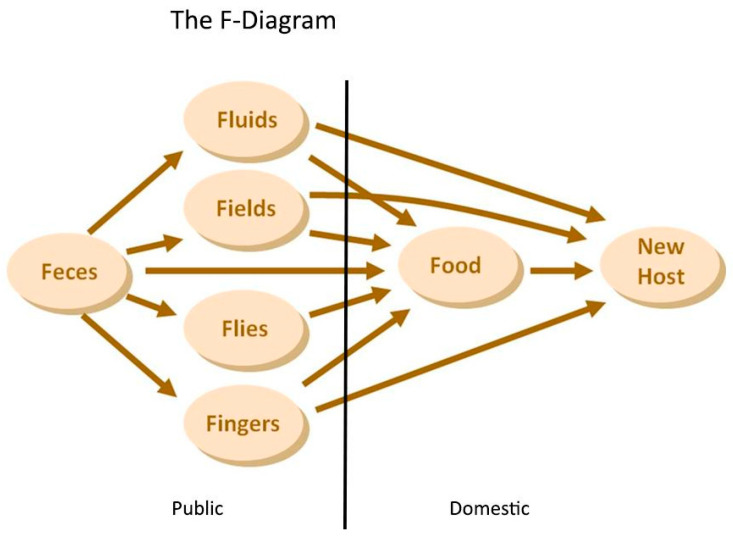
F-diagram divided into public and domestic domains.

This paper will depart from the F-diagram to investigate whether the missing apparent connection between water quality and health is simply caused by methodological problems or by an actual lack of causality. However, to fully utilize its potential for transmission route analysis and to illustrate the points where we physically currently assess the routes, the F-diagram should be divided into the two spatial domains (Figure 1), as suggested by Cairncross et al. [20]—the public domain and the domestic domain.

The public domain refers to locations outside the household, such as public water sources, markets, fields, etc., from which pathogens are imported into the domestic domain.

The domestic domain pertains to the areas inside household compounds, where pathogens are exchanged/recirculated between the kitchen, latrine, etc., by persons, domestic animals, or vectors such as flies and cockroaches.

To quantitatively investigate the flow of the pathogen in the F-diagram, water and food quality has traditionally been monitored in the public domain—at the point-of-delivery (water tap) or at the shop/market (for food quality)—using *Escherichia coli* as the fecal indicator organism. Still, some studies and guidelines have also included monitoring *E. coli* in the domestic domain, in drinking water containers (point-of-use) or cooked food [21]. However, from our previous research in a low-income area in Dhaka, Bangladesh, we found that the *E. coli* contamination identified in the household’s drinking water containers is not always a good indication of the quality of the water actually being drunk, i.e., the quality of the water inside the drinking vessel (cup, glass, or jar). In addition, in our previous investigations, we did not find any significant evidence connecting the water quality inside the drinking vessel to any pretreatment of the water (boiling or filtering) in the domestic domain [22]. Further, we found that the *E. coli* content in the food eaten might also significantly contribute to the total daily *E. coli* ingestion, far exceeding the amount of *E. coli* ingested via the drinking water [23].

To further investigate pathogen transmission in the domestic domain, its influence on household hygiene assessments, and the implications for future WASH projects, this paper analyzes the *E. coli* contamination of fomites in identified problematic hygiene hotspots inside low-income households in Dhaka. Fomites (also called passive vectors) are objects or surfaces that are likely to carry infection, such as door handles, clothing, or kitchen utensils. By analyzing the *E. coli* contamination on fomites in low-income households, this paper aims to shed light on not only the pathogen flow inside the domestic domain, but also the potential role of fomites on the transmission of pathogens responsible for diarrheal disease in low-income settings.

## 2. Materials and Methods

This paper represents a sub-study performed under the more extensive longitudinal study “Combating Cholera Caused by Climate Change, C5”. Field staff from the International Center for Diarrheal Disease Research, Bangladesh (icddr,b), conducted community visits and sample collection. Sample processing and microbiological analysis were performed at the University of Dhaka. Study protocols have been previously published [24].

### 2.1. Study Area

East Arichpur, Tongi, is located on the outskirts of greater Dhaka. Several industrial establishments and garment factories surround the area; consequently, this 1.2 km^2^ area is densely populated with approximately 120,000 people in 29,000 low-income households, mostly with one nuclear family per room. The houses are predominately one-story, with occasional multi-story buildings in the area. Typically, 10–15 small rooms are situated around a yard with shared common cooking areas, latrines, and water supplies [24]. The water in this area is supplied by two types of communal pumps: the “WASA (Water Supply and Sewerage Authority) pump”, installed by the municipal government and connected to public tapstands through underground networks of pipes, and/or the “submersible pump”, installed by individuals or groups of residents and connected to shared multi-household tapstands through above-ground networks of pipes [22]. Kitchen tables are not used in this community, and vegetables, fish, and meat are cut on the kitchen floor with a large wooden mounted sickle-shaped metal knife called a “Boti.” After cutting, the food is kept on a plate, and the waste is left on the floor. Pots, plates, glasses, and utensils are usually washed under a tap after use (sometimes washed in a bucket, depending on the household’s water supply) and left to dry. Most people use their hands to eat, and the food is served from a shared plate. As many women work long hours in the garment industry and, therefore, only have time to cook once a day, households often eat food prepared several hours beforehand and stored outside at room temperature.

**Figure 2:**
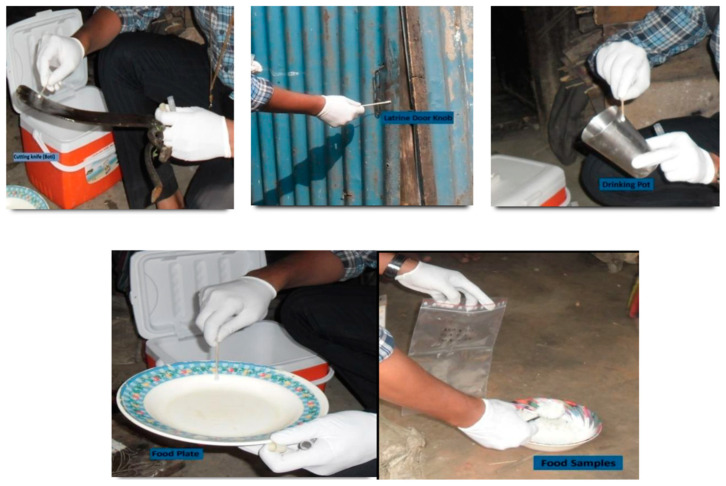
Sample collection from different surface locations and food.

A total of 32 households were selected out of the 577 households participating in the C5 study. A baseline survey included hygiene observations by an anthropological team on hand contact frequency with objects in each household, including kitchen and sanitation facilities. After this survey, four main contamination hotspots (or fomites) of high-frequency direct exposure were selected within the household (domestic) domain: (1) the traditional cutting knife, or “Boti”; (2) cleaned plates used to serve the meal; (3) latrine door knobs; and (4) the inner surfaces of drinking water vessels (i.e., mugs, glasses, bottles, jugs, or pitchers) from which household members drink water (Figure 2). Hotspots were determined as places where bacteria can potentially be transmitted from one person to the other or which “receive” bacteria from the external environment. Specifically, hotspots are surfaces that are suspected to be contaminated with fecal material and/or raw fish, chicken, or any kind of meat due to either frequent touching, infrequent cleaning, and/or poor kitchen or personal hygiene.

A routine visit to each household was conducted every six weeks between June 2014 and December 2015, where a swab sample was taken from each “hotspot”. A total of 668 environmental swab samples or “hotspot” samples were collected. The samples were obtained by swabbing an area of approximately 10 cm^2^ with a sterile cotton swab moistened in phosphate buffer saline (PBS). The swab was then immersed in a tube containing 3 mL PBS. All samples were maintained at 4 °C in a sterile container and transferred to the Environmental Microbiology Laboratory, University of Dhaka, within 4 h of collection [24]. Hotspot swabs in PBS solution were vortexed for 1 min; then, 100 µL of the supernatant was inoculated on modified thermotolerant *E. coli* (mTEC) agar plates (m-TEC Chromo Select Agar, SIGMA-ALDRICH, St. Louis, MO, United States). After incubation at 44.5 °C for 24 h, enumeration of thermo-tolerant *E. coli* was performed with a detection limit of 1 cfu/10 cm^2^.

At the household visits, and as part of the more extensive C5 study, additional information on water usage was collected, and water samples were collected at the “point-of-drinking” (glasses, mugs, bottles, or jugs used by household members to drink water) and from the public domain (the communal-source tap stands used by the household). The water samples from the sources were taken directly from taps attached to the communal pumps used by each studied household. Water samples of 150–200 mL were collected using pre-sterilized wide-mouth sampling bottles (SPL Life Sciences, Gyeonggi-do, Republic of Korea) and transported to the Environmental Microbiology Laboratory, University of Dhaka, within two to four hours of collection. Microbiological water quality was assessed using standard membrane filtration and culture methods for the detection of *E. coli*, and 100 mL aliquots of the water samples were filtered through white gridded membrane filters with pore sizes of 0.45 µm and 47 mm in diameter (S-Pak, Merck Millipore, Darmstadt, Germany). Membranes were placed on plates of modified Thermotolerant *E. coli* agar (m-TEC agar, Oxoid, London, UK) and incubated at 44.5 ± 0.5 °C for 18–24 h. Typical reddish-purple or magenta colonies of *E. coli* were enumerated and recorded as colony-forming units (cfu) per 100 mL of water [25].

The *E. coli* concentration between communal source water and point-of-drinking water were paired for each household, and the difference calculated to determine changes in water quality within the same day. The difference was calculated by deducting the *E. coli* concentration of communal sources from the *E. coli* concentration of the point-of-drinking water. The full methodology and results were previously described by Ferdous et al. [22].

### 2.2. Ethical Clearance

Informed written consent was obtained from all study households. This study was approved by the Institutional Review Board (IRB) of icddr,b.

## 3. Results

The baseline survey on hygiene behavior revealed that in 6 out of 32 households, the plates and utensils used for the next meal were visibly dirty (e.g., visible dust, grime, stains of food). However, all food plates swapped for this study had been cleaned after the previous meal with or without cleaning materials, such as dishwashing soap, and were primarily dry. The “Boti” cutting knife was usually not visibly dirty at the time of swab collection, as the knife was continuously used for daily cooking.

Despite being washed, the highest average contamination was found on the food plates, at 253 cfu/10 cm^2^, closely followed by the Boti, with 240 cfu/10 cm^2^ (Table 1), which also had the highest single *E. coli* count of 2880 cfu/10 cm^2^. The latrine doorknob had the lowest contamination, with an *E. coli* mean of 73 cfu/10 cm^2^. Finally, the mean *E. coli* contamination measured on the surface of the drinking water vessels (point of drinking) was 167 cfu/10 cm^2^. The monthly prevalence of *E. coli* on the surface swabs showed no specific seasonal patterns, although our water-use monitoring showed a decreased level of water use in the cold season [19].

As to the microbiological quality of water samples, *E. coli* was detected in 58% of the communal source water samples and in 77% of the point-of-drinking samples. *E. coli* contamination at the point of drinking was higher than contamination at the communal source in 51% of samples. Detailed results on the microbiological water quality were previously published by Ferdous et al. [22].

## 4. Discussion

Ideally, *E. coli* bacteria should not be found in the kitchen environment nor in drinking water. Still, this is far from the reality in a low-income household in Dhaka. This study found substantial contamination within the kitchen environment, with the cutting knife and washed food plates being more contaminated than the drinking vessels and, even the latrine doorknob. These results provide new insights into the potential transmission routes. For example, eating freshly cooked rice, which we normally consider hygienically safe, if served on a newly washed food plate, can suddenly constitute a health concern, as can pre-treated/boiled drinking water drunk from a newly washed glass.

### 4.1. Drinking Water Contamination in the Domestic Domain

In-house contamination of drinking water is not a new discovery. Several studies have documented the contamination in the household water storage container to be unlinked to the quality of the public water source [26,27]. However, in addition to the expected contamination between the public source and the in-house water storage container, we also observed contamination between the water storage containers (point-of-use) and the point-of-drinking water [22]. This discovery of the potential interdependency of the water quality between point-of-use and point-of-drinking implies that a sample taken from a household storage container does not necessarily correlate with the actual pathogen load to which a person is exposed. Domestic domain water quality interventions have normally focused on providing a safe water storage unit, protecting the drinking water from human contact in the domestic domain at the point-of-use [26]. With the documentation of point-of-drinking contamination, our focus may need to be bifocal, both on storage containers and on drinking vessels. However, in households with high levels of kitchen hygiene, the point-of-drinking contamination would be expected to be less important, as it most likely originates from the lack of proper dishwashing, cross-contamination, or storage of drinking vessels. In Dhaka, Pickering et al. [28] saw a positive effect on childhood diarrhea after chlorinating the household water at the point of collection in the public domain. However, it can be speculated as to whether this was caused by the bactericidal effect of free residual chlorine in the domestic water used for kitchen hygiene, or due to the free residual chlorine in the drinking vessel, which potentially caused low contamination levels at the point-of-drinking.

### 4.2. Kitchen and Food Contamination

Keeping good food hygiene standards in a kitchen with no running water, no kitchen table, no refrigeration, and no safe food storage can be challenging [29], and previous analyses of surfaces within the study households clearly illustrate this [30]. In the daylong hygiene observations of the C5 study [31], it was observed that the Boti was used for every cutting purpose in the food preparation process (without cleaning). As a result, it was able to efficiently transfer contaminants from one food type to another. Further, another C5 study showed that, when investigating Hilsha fish’s ability to transfer *Vibrio cholerae* bacteria, a scattering of fish slime (containing both *E. coli* and *Vibrio cholerae* bacteria) and fish scales was closely linked to the use of the Boti [31], and contaminated the rest of the kitchen environment.

In a separate C5 study, we reported that 50% of the flies landing on a bowl of freshly cooked rice in the area (an average of 90 sec between landings) would deposit more than 600 *E. coli* per landing [32]. This top contamination from flies and the bottom contamination from the washed plates would give the freshly cooked rice a considerable dose of contamination. For instance, in the project area, we estimated that by eating food stored uncovered for 6 h, 94% of an average person’s total daily intake of *E. coli* would come from food alone and only 6% from drinking water [23]. This estimate considered a daily consumption of 1.8 L of polluted untreated drinking water with contamination of 98 *E. coli* per 100 mL [23]. This value was the mean for all 211 water samples taken at the point-of-drinking from the same households [33].

Contamination of glasses and plates can originate from different sources. Polluted washing/rinsing water would intuitively be the most obvious (not measured). Still, a median contamination of 98 *E. coli* per 100 mL of the domestic water [23] would not accommodate such a concentration as that seen on the washed plates. However, considerable domestic contamination of the washing water could be expected, as the washing/rinsing often was performed in a bucket with reused water combined with sources such as contaminated hands or fly deposits. Sultana et al. [19] showed that the quantity of the water used for kitchen hygiene and especially washing water in the household would be reduced when the household was under water stress. To complicate possible interventions further, we used new genetic methods that indicated that 85% of the pathogenic *E. coli* found at the point-of-drinking could have originated from animals and birds [25]. However, this would need to be further investigated, as it might challenge the traditional F-diagram notion of transmission routes.

### 4.3. Unfolding the Domains

**Figure 3:**
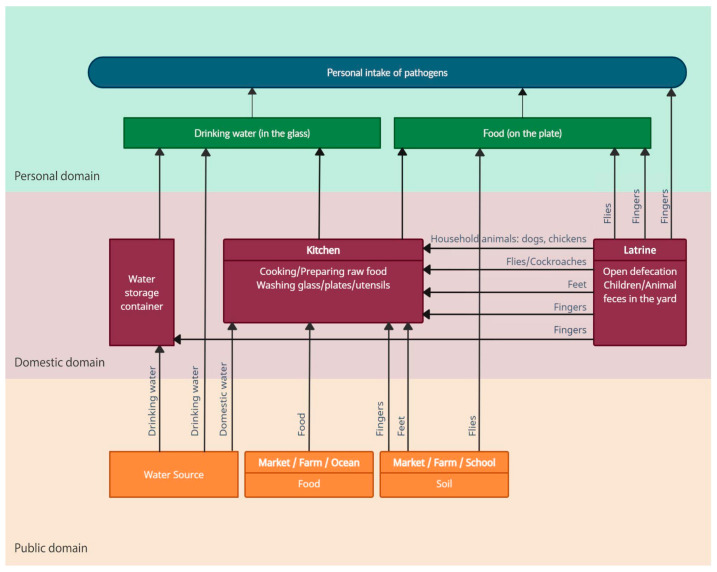
Transmission routes in the public, domestic, and personal domains in a household in Arichpur, Dhaka.

The discussion above clearly indicates that we have difficulty assessing and understanding contamination routes. If we wish to obtain a clear understanding of the relative importance of the different contamination routes, we need to develop a new methodology regarding how (or where) to measure the correct pathogen flow in each of the transmission routes. With this, we can evaluate where possible interventions would have the most significant impact, or where no effect can be expected. For this purpose, the two-domain model is insufficient to capture the actual pathogenic load consumed by a person. Therefore, this paper proposes that a third domain, “the personal domain”. i.e., the immediate sphere around the person covering ingestion of water from a dirty glass, food from a contaminated plate, or fingers inserted into the mouth, should be added alongside the public and domestic domain. An example of a household transmission model used on a Bangladeshi household with the three domains incorporated is illustrated in Figure 3.

The assessments in the personal domain will enable us to address the true exposure at the time of consumption and, as a result, to better assess and evaluate the potential of different interventions and, with this, potentially clarify why historical interventions did not have the expected impact.

**Figure 4:**
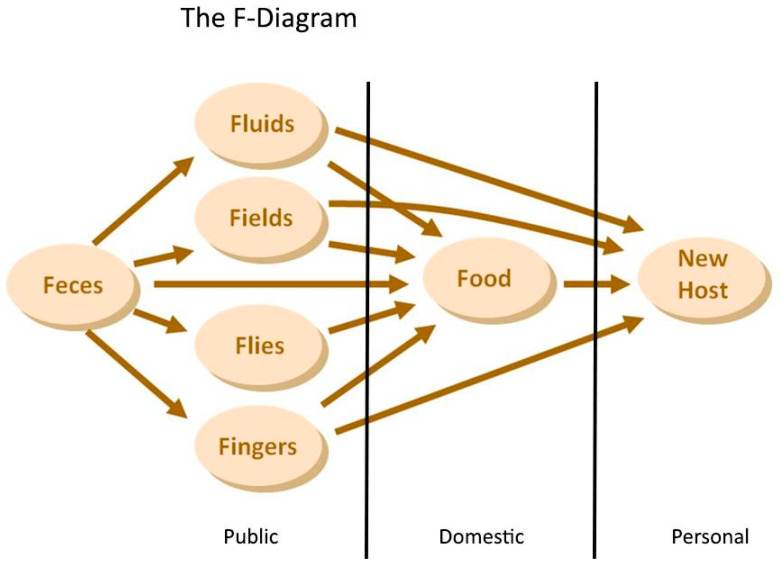
Public, domestic, and personal domains incorporated into the traditional F-diagram.

Due to the complexity of the transmission routes in the domestic domain, we only had the chance to assess the true pathogen exposure by measuring the pathogen flow in the personal domain. By assessing the pathogen flow in the personal domain and investigating the flows “upstream” of the domestic domain (Figure 3), as well as, eventually, the public domain, we can evaluate which interventions might have an effect and which ones will be diluted in terms of overall exposure (Figure 4).

This study only included 32 households; thus, the results might be slightly different than if the sample size were larger. However, the trend of in-house contamination will most likely be maintained, as this is in line with the trend that we have observed throughout the C5 study [34]. More extensive studies with higher statistical power are needed to investigate whether this contamination in the personal domain is limited to *E. coli* only, or whether it can be expanded to other bacteria, viruses, or even protozoa.

Further, additional intervention studies are needed to investigate this new domain and pinpoint new and unknown transmission routes that have yet to be noticed as we turn the focus from the public and domestic domains to the personal domain.

## 5. Conclusions

In conclusion, the personal domain assessment should be considered the starting point in any WASH investigation. Our study results have shown that a significant amount of contamination exists on both food plates and utensils, even if all have previously been washed. Hence, these sources constitute the primary means of *E. coli* exposure in the studied households. This high level of contamination could be attributed to several factors, most likely in combination, such as inadequate cleaning practices, water quality, or food handling practices. Further investigations are required to determine the exact cause of the high levels of contamination. Nevertheless, exposure to pathogens within the household environment appears to occur primarily at the point of consumption, or the personal domain, which can include direct contact with fingers, dirty glasses, or contaminated plates.

Traditionally, WASH investigations and interventions are conducted within the public and domestic domains. Our results highlight the importance of evaluating the hygiene behavior and practices of households not only in the public and domestic domains, but also within the immediate sphere around a person—the personal domain.

The introduction of the personal domain assessment allows us to evaluate the true pathogen flows in the domestic hygiene environment more accurately. It offers a more detailed understanding of the different exposure routes, enabling us to identify the routes for which breaking is most likely to have the greatest impact.

We argue that the introduction of the personal domain in WASH investigations can provide a more holistic view of the household’s hygiene practices, enabling us to develop more effective interventions that target the specific transmission routes, and, consequently, further reduce the risk of pathogen exposure in the household.

In summary, our study highlights the importance of evaluating household hygiene practices within a new sphere—the personal domain. The personal domain assessment should serve as the foundation of any WASH investigation, guiding our efforts towards more effective and sustainable solutions. If we only measure contamination at the domestic domain level, we will likely miss the overall picture and risk repeating yesterday’s interventions with minimal effect on the livelihoods of the people most at risk.

*Life can only be understood backwards, but it must be lived forwards*. Soren Kierkegaard, 1843.

## Figures and Tables

**Table 1:** *E. coli* contamination of hotspots within the household environment.

Hotspot Location	N	*E. coli* Count (cfu/10 cm^2^)		
		Median	Mean	Confidence Interval (95%)
Food plate	165	30	253	186.06–319.45
Cutting knife (Boti)	169	18	240	171.97–308.65
Point of drinking/drinking vessel surface	169	0	167	114.76–219.7
Latrine doorknob	169	0	73	38.68–107.65
